# Health Tract, No. 123

**Published:** 1865

**Authors:** 


### POTATOES.

The proper cooking of good food is an essential element of good health in all civilized countries. The general use of the potato shows that it is palatable and healthful; but few families in this country fail to have it on the table once a day. In Ireland, it is the chief article of food at every meal, and it is said that there are multitudes who seldom eat any thing else. It has the same amount of nutriment as the egg, thirteen per cent; it has twice the nutriment of coffee; half as much as beef. It requires two hours and a half for digestion, raw cabbage two hours, roast beef an hour longer, roast pork an hour longer still. It is claimed that the outer quarter of an inch of the potato contains more nourishment than the entire remainder. Hence peeling is a waste. They should be cooked, then the very thin skin is easily removed, and the whole nourishment remains. Late in the spring, as the potato prepares for sprouting, the outer portion becomes "rank," and it is better to peel before cooking. If kept in a dark place, sprouting is much retarded, and further if the sprouts are rubbed off with the hands. The lighter a potato is, the more mealy and palatable it will be after cooking; hence the good ones float, while others sink in strong salt-water. Boiled potatoes are not digested so easily or so soon as if baked or roasted.

Cooking Potatoes.—They should be well washed and put into cold water with the skin on. Gradually heat the water, and when near boiling, add more cold water; if thus checked, the skins will not crack until the potato is thoroughly done; pour off the water, and let the skins become dry before peeling. The Irish nick out a piece of the skin before putting them in the pot. The potatoes of each cooking should be nearly the same size, that all may be equally done. They should not be covered with more than an inch of water, that they may be just covered at the finish; they will become waxy and watery if allowed to remain in the water a moment after they are well done. After they are dried they can be kept hot and mealy for some time, if covered with a a napkin of the diameter of the containing vessel. This is better than steaming, and they are prepared in half the time. Moderate-sized potatoes should be done enough in a quarter of an hour. They sprout least in the darkest places.

Cold Potatoes Fried.—Put a bit of cream-dripping into a frying-pan; when it is melted, slice in your potatoes with a little pepper and salt; put them on the fire; keep stirring them; when they are quite hot, they are ready.

Potatoes Mashed.—When your potatoes are thoroughly boiled, drain them quite dry, pick out every speck, etc., and while hot, rub them through a colander in a clean stew-pan. To a pound of potatoes put about half an ounce of butter and a table-spoonful of milk; do not make them too moist; mix them well together.

Potatos Mashed with Onions.—Prepare some boiled onions by putting them through a sieve, and mix them with potatoes. In proportioning the onions to the potatoes, you will be guided by your wish for more or less of their flavor.

Potatoe-Flour and Jelly.—Rasp the potatoes into a vessel of cold water, and change it frequently, until the raspings fall to the bottom like a paste, then dry in the air, pound in a mortar, and pass it through a hair-sieve. This is nearly as nutritive, and lighter than flour; hence is better for pastry and puddings for invalids. If kept dry, it will remain good for years, while it is easily converted into a most nutritious jelly, by pouring absolutely boiling water on it. When changed into jelly, flavor to taste, and use it.

To Brown.—While the meat is roasting, and an hour before serving, boil the potatoes, take off the skin, flour them well, put them under the roasting meat, and let them drip before going on the table.

To Roast.—Clean well, nick out a small piece, and roast A little butter over the skin crisps them.

*Cold Potatoes*, boiled for dinner, and left over, make an excellent dish for breakfast, by covering them with milk or cream in a frying-pan; add butter and salt, and let remain until the milk thickens—say fifteen minutes.

Potatoes, when boiled, if either waxy or to be eaten with cold meat, should be peeled and put whole on the gridiron until nicely browned.

For Stews.—Potatoes should be always boiled a little before putting into into stews, as the first water is a little poisonous. Fried potatoes may be cut from raw, half an inch thick; fry quickly in hot fat, let grease drip off, dry, salt and use.

Keeping Potatoes.—If laid on straw on the ground, and covered with straw and then a layer of earth a foot deep, they will produce shoots near the end of spring; if two feet, shoots appear at midsummer; at six feet they cease to vegetate, and will keep for two or more years in a perfect state. There should be a trench a foot deep around the pile, unless the soil is very sandy.

